# Subarachnoid hemorrhage triggers neuroinflammation of the entire cerebral cortex, leading to neuronal cell death

**DOI:** 10.1186/s41232-022-00236-4

**Published:** 2022-12-14

**Authors:** Hiroki Yamada, Yoshitaka Kase, Yuji Okano, Doyoon Kim, Maraku Goto, Satoshi Takahashi, Hideyuki Okano, Masahiro Toda

**Affiliations:** 1 Department of Neurosurgery, Keio University School of Medicine, 35 Shinanomachi, Shinjuku-ku, Tokyo, 160-8582 Japan; 2 Department of Physiology, Keio University School of Medicine, 35 Shinanomachi, Shinjuku-ku, Tokyo, 160-8582 Japan; 3 The University of Tokyo, 7-3-1 Hongo, Bunkyo-ku, Tokyo, 113-8655 Japan

**Keywords:** Cerebral cortex, Early brain injury, Microglia, Neural cell death, Neuroinflammation, Subarachnoid hemorrhage (SAH)

## Abstract

**Background:**

Subarachnoid hemorrhage (SAH) is a fatal disease, with early brain injury (EBI) occurring within 72 h of SAH injury contributes to its poor prognosis. EBI is a complicated phenomenon involving multiple mechanisms. Although neuroinflammation has been shown to be important prognosis factor of EBI, whether neuroinflammation spreads throughout the cerebrum and the extent of its depth in the cerebral cortex remain unknown. Knowing how inflammation spreads throughout the cerebrum is also important to determine if anti-inflammatory agents are a future therapeutic strategy for EBI.

**Methods:**

In this study, we induced SAH in mice by injecting hematoma into prechiasmatic cistern and created models of mild to severe SAH. In sections of the mouse cerebrum, we investigated neuroinflammation and neuronal cell death in the cortex distal to the hematoma injection site, from anterior to posterior region 24 h after SAH injury.

**Results:**

Neuroinflammation caused by SAH spread to all layers of the cerebral cortex from the anterior to the posterior part of the cerebrum via the invasion of activated microglia, and neuronal cell death increased in correlation with neuroinflammation. This trend increased with the severity of the disease.

**Conclusions:**

Neuroinflammation caused by SAH had spread throughout the cerebrum, causing neuronal cell death. Considering that the cerebral cortex is responsible for long-term memory and movement, suppressing neuroinflammation in all layers of the cerebral cortex may improve the prognosis of patients with SAH.

**Supplementary Information:**

The online version contains supplementary material available at 10.1186/s41232-022-00236-4.

## Introduction

Subarachnoid hemorrhage (SAH) is a serious disease of the cerebral vasculature that is primarily caused by a ruptured cerebral aneurysm [1]. Approximately 6 to 9 people per 100,000 are affected by SAH every year [2]. Although SAH accounts for 5–10% of all strokes [3], its mortality rate is as high as 50%. In addition, SAH occurs not only disproportionately in the elderly but also frequently in subjects of mature age, resulting in significant social and medical economic losses [4, 5].

Therefore, the mechanisms and prognostic factors of SAH urgently need to be elucidated to develop fundamental therapeutic strategies. Recently, the concept of early brain injury (EBI) has emerged as an important prognostic factor [6]. EBI is a term used to describe the pathological event occurring within 72 h of the onset of SAH [7], which is known to be caused by a variety of factors, including mechanical damage [8], disruption of vascular integrity [9, 10], hippocampal damage [11], and various molecular changes [12, 13].

Neuroinflammation is known to be an important factor of EBI [14, 15]. Red blood cells that enter the subarachnoid space upon aneurysm rupture are degraded to release free heme, which promotes oxidative stress and activates the inflammatory cascade, resulting in neuroinflammation [16, 17]. Additionally, microglial activation and neuroinflammation are involved in the development and recovery of disease complications [18]. This neuroinflammation leads to neuronal cell death, which increases the degree of disability [19].

Several reports have focused on specific areas of the brain, such as the ventral cerebral cortex near the hematoma [15], which is most affected by pressure damage due to aneurysm rupture, and the hippocampus [11], which is vulnerable to ischemia. However, it is not clear whether the damage caused by neuroinflammation is confined to these regions or extends to the entire cerebrum.

In addition, to assess the extent of neuroinflammation, mouse models with varying degrees of disease severity are needed. However, only a few studies have reported these analyses, and it was not clear how the degree of disability caused by EBI varied with the severity of the disease.

Here, we created a mouse model of SAH according to the severity and clarified which regions of the cerebrum were affected by neuroinflammation-induced neuronal cell death and to the extent to which the degree of damage differed depending on the severity. The results of this study may help to further elucidate the pathogenesis of SAH and appropriate therapeutic strategies by extensively examining the regions affected and the disease severity.

## Results

### Creation and evaluation of mouse models of SAH of varying severity

**Fig. 1:**
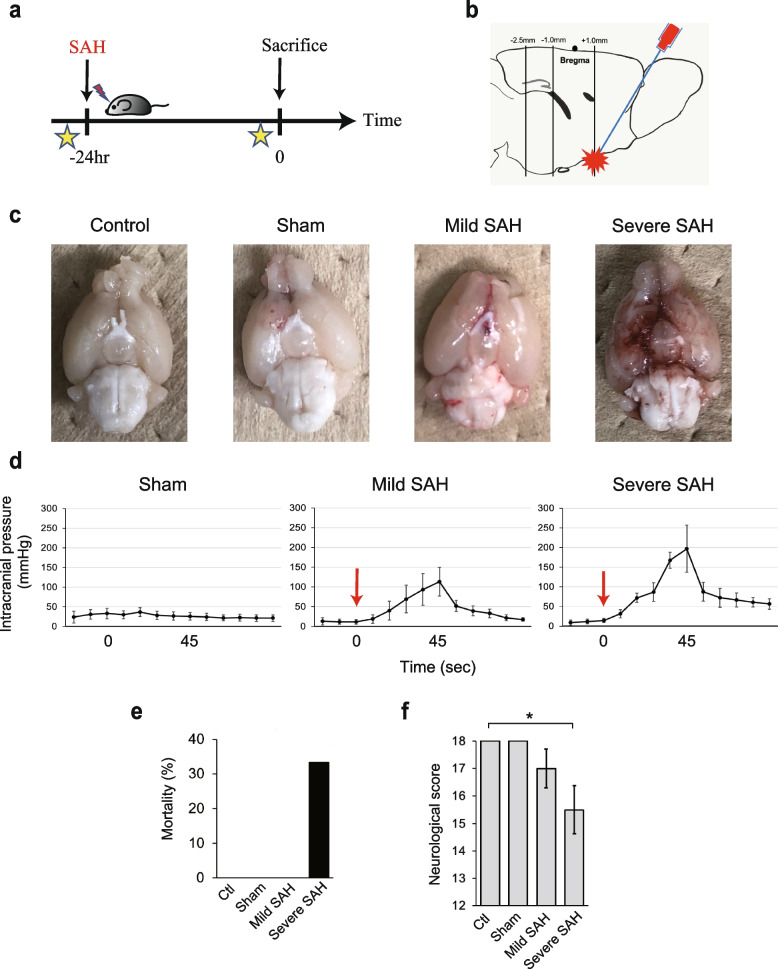
An experimental SAH model by severity. **a** Simplified diagram of the experiment. In the SAH group, SAH was induced by blood injection 24 h before sacrifice. The neurocognitive score (modified Garcia score) was evaluated just prior to SAH induction and sacrifice (star). **b** Schematic diagram of the site of blood injection to create the subarachnoid hemorrhage model. **c** Photographs of the brains of each mouse after sacrifice showing no hematoma in the control and sham groups and increased hematoma volumes in the severe SAH group compared to the mild SAH group. **d** Intracranial pressure in each mouse during surgery. In the SAH groups, the hematoma was injected (red arrow) over 45 s, and the intracranial pressure gradually increased. The peak intracranial pressure was the highest in the severe SAH group. **e** Mortality of mice in the control, sham, and SAH groups (*n* = 18, total). **f** Modified Garcia score at 24 h is shown for each group. The severe SAH group had significant decreased neurological scores compared to those of the control group. Statistical analysis was performed with an unpaired two-tailed Student’s *t* test. The values in the bar graphs represent the mean ± SD. *p < 0.05

We created both mild and severe SAH mouse models by combining two indicators: the volume of the injected hematoma and the neurological scores at 24 h after SAH (Fig. 1a, b). Twenty-four hours after prechiasmatic cistern blood injection, SAH was confirmed to have occurred in the brain following fixation (Fig. 1c). The intracranial pressure at the time of the procedure did not increase with the sham model, whereas it increased to an average of 113.0 (± 36.6) mmHg at peak (after 45 s) in the mild SAH model and to 196.8 (± 60.1) mmHg in the severe SAH model (Fig. 1d).

The mortality rate of mice was 0% in the negative control (not to be operated), sham, and mild SAH groups, and 33% in the severe SAH group (Fig. 1e). The modified Garcia score (Supplementary Table 1) at 24 h after the procedure was 18 for all mice in the control and sham groups; 16, 17, 17, and 18 (*n* = 4) for mice in the mild SAH group; and 14, 16, 16, and 16 (*n* = 4) for mice in the severe SAH group (Fig. 1f).

### Neuroinflammation caused by SAH alters the expression of microglia-specific genes

EBI, which develops within 72 h after SAH injury, induces inflammation that leads to neurological damage [20]. However, it is not known how far inflammation extends into the cerebral cortex. First, we reanalyzed the RNA sequencing (RNA-seq) data of an SAH model mouse analysis study [21] to determine which inflammatory markers should be used to analyze the extent of inflammation spillover using the cerebral cortices of SAH model mice in detail.

**Fig. 2:**
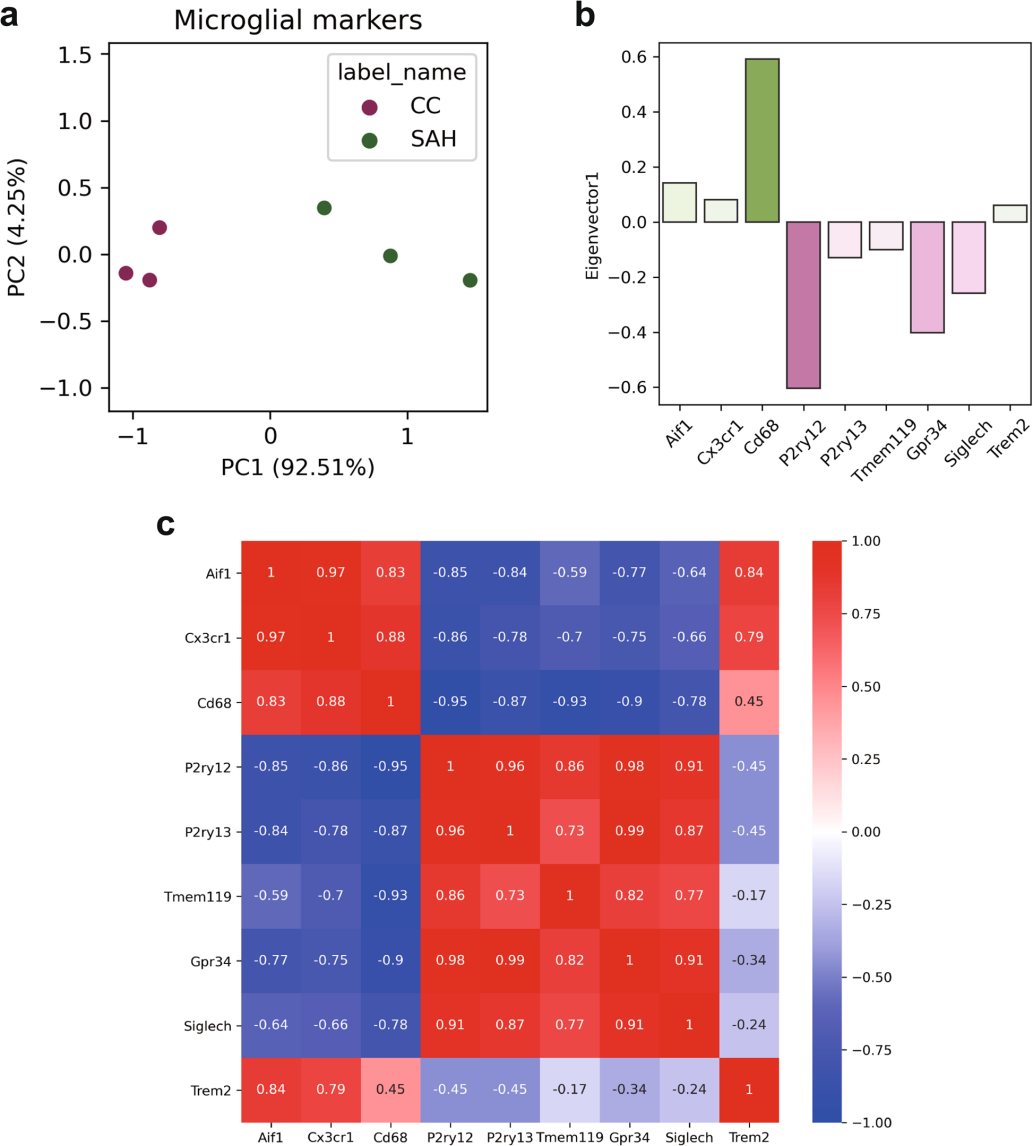
Expression patterns of microglial markers. **a** A scatterplot of microglial marker expression. The expression matrix of the microglial markers (Aif1, Cx3cr1, Cd68, P2ry12, P2ry13, Tmem119, Gpr34, Siglech, and Trem2) was decomposed into principal components (PC1, 2, … n, where n is the rank of the matrix). The horizontal axis represents the PC1 score, and the vertical axis represents the PC2 score. **b** The four genes (Aif1, Cx3cr1, Cd68, Trem2) had positive loadings in eigenvector 1, which suggested that the PC1 score increased as the expression levels of the four genes increased. **c** A heatmap of the correlation matrix of microglial marker expression

To determine whether the expression patterns of the microglial markers varied between the control and SAH groups, we examined a set of RNA-seq data acquired under desirable conditions (GSE79416). When we quantified the expression patterns of notable microglial markers (*Aif1*, *Cx3cr1*, *Cd68*, *P2ry12*, *P2ry13*, *Tmem119, Gpr34*, *Siglech*, and *Trem2*) by principal component analysis, overt gaps in the PC1 scores were observed between the SAH group and the controls (Fig. 2a). This result was interpreted as the differences in the expression levels of microglial markers because certain genes, including *Aif1* (a coding gene of Iba1 protein), have a positive loading on eigenvector 1, while the rest have negative loading (Fig. 2b) [22]. Since *Aif1* was highly correlated with other upregulated genes, *Cx3cr1*, *Cd68*, and *Trem2*, we used *Aif1* as an indicator of microglial markers induced in SAH (Fig. 2c)*.*

On these grounds, we focused on microglia [18] induced by neuroinflammation and used Iba1 [23] as a marker of microglia. Iba1-positive cells with radially extending projections are known as activated microglia [24], which are induced by inflammation, and their numbers were quantified in each brain section.

### In the mouse model of SAH, neuroinflammation spills over into the deep VI layer of the cerebral cortex in both the anterior and posterior regions of the cerebrum

As mentioned above, we constructed model mice according to the severity of SAH and sectioned their brains 24 h later (Fig. 1a) to compare the numbers of active microglia in the control, sham, mild SAH, and severe SAH groups. In addition, sections from three regions (1.0 mm anterior to bregma, 1.0 mm posterior to bregma, and 2.5 mm posterior to bregma) were prepared and analyzed (Fig. 1b).

**Fig. 3:**
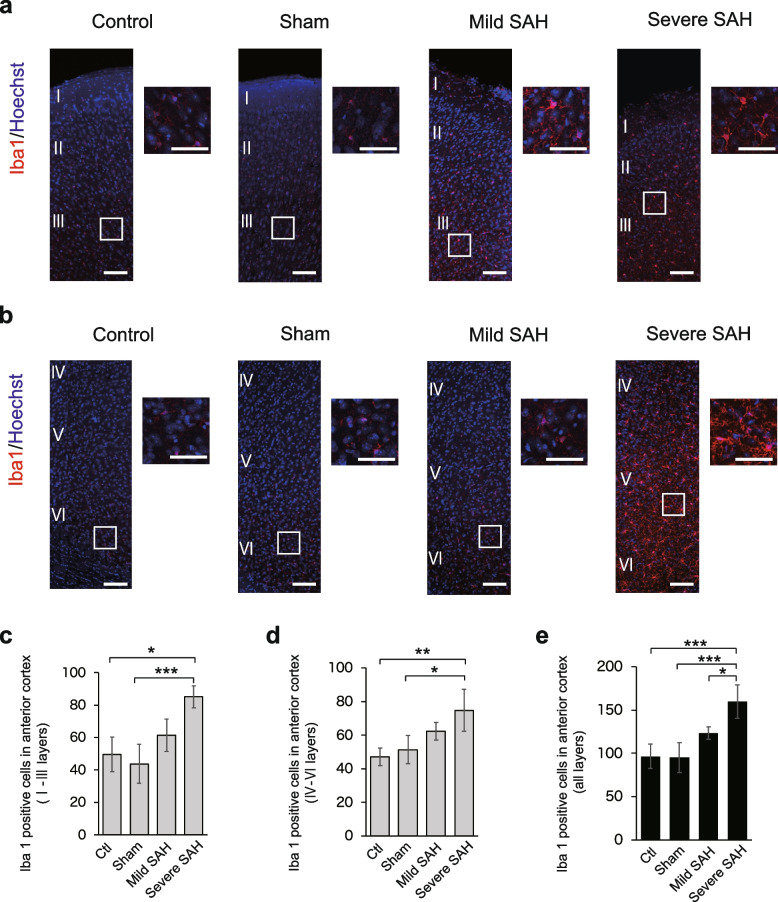
Iba1-positive microglia infiltrated layers I to VI of the cerebral cortex (dorsal region) located in the anterior region of the brain (1.0 mm anterior to bregma). **a** Representative immunostaining images of Iba1-positive cells (red) in layers I to III of the anterior cerebrum in the control, sham, mild SAH, and severe SAH groups. Scale bar, 100 μm for the general figure and 50 μm for the enlarged figure. **b** Representative immunostaining images of Iba1-positive cells (red) in layers IV to VI of the anterior cerebrum in the control, sham, mild SAH, and severe SAH groups. Scale bar, 100 μm for the general figure and 50 μm for the enlarged figure. **c** Quantification of the number of Iba1-positive cells in layers I to III of the anterior region of the cerebrum (*n* = 4, each group, control vs. severe SAH; *p* = 0.002, sham vs. severe SAH; *p* < 0.001). **d** Quantification of the number of Iba1-positive cells in layers IV to VI of the anterior region of the cerebrum (*n* = 4, each group, control vs. severe SAH; *p* = 0.003, sham vs. severe SAH; *p* = 0.01). **e** Quantification of the number of Iba1-positive cells in all layers of the anterior region of the cerebrum (*n* = 4, each group, control vs. severe SAH; *p* < 0.001, sham vs. severe SAH; *p* < 0.001, mild SAH vs. severe SAH; *p* = 0.025). Statistical analyses were performed with one-way ANOVA and Tukey–Kramer post hoc tests. The values in the bar graphs represent the mean ± SE. *p < 0.05, **p < 0.01, ***p < 0.001

In the anterior part of the cerebrum (1.0 mm anterior to bregma), the number of Iba1-positive microglia increased with the severity of SAH in layers I to III (superficial layers) of the dorsal region of the cerebral cortex (Fig. 3a, c). The number of Iba1-positive microglia increased with the severity of SAH in the deeper layers IV to VI (Fig. 3b, d). In the anterior cerebral region of the SAH model mice, active microglia were induced in all layers of the cerebral cortex (Fig. 3e), indicating that neuroinflammation spread throughout all layers.

**Fig. 4:**
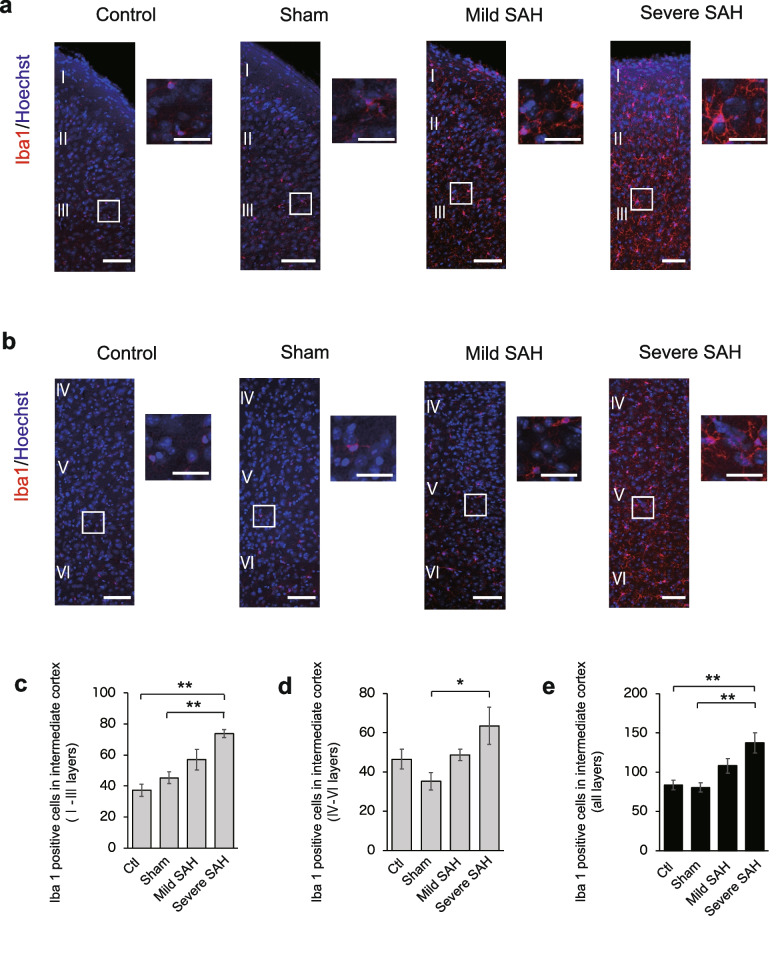
Iba1-positive microglia infiltrate the intermediate cortex in the SAH mouse model (1.0 mm posterior to bregma). **a** Representative immunostaining images of Iba1-positive cells (red) in layers I to III of the intermediate cerebrum in the control, sham, mild SAH, and severe SAH groups. Scale bar, 100 μm for the general figure and 50 μm for the enlarged figure. **b** Representative immunostaining images of Iba1-positive cells (red) in layers IV to VI of the intermediate cerebrum in the control, sham, mild SAH, and severe SAH groups. Scale bar, 100 μm for the general figure and 50 μm for the enlarged figure. **c** Quantification of the number of Iba1-positive cells in layers I to III of the intermediate region of the cerebrum (*n* = 4, each group, control vs. severe SAH; *p* = 0.001, sham vs. severe SAH; *p* = 0.008). **d** Quantification of the number of Iba1-positive cells in layers IV to VI of the intermediate region of the cerebrum (*n* = 4, each group, control vs. severe SAH; *p* = 0.230, sham vs. severe SAH; *p* = 0.025). **e** Quantification of the number of Iba1-positive cells in all layers of the intermediate region of the cerebrum (n=4, each group, control vs. severe SAH; *p* = 0.005, sham vs. severe SAH; *p* = 0.003). Statistical analyses were performed with one-way ANOVA and Tukey–Kramer post hoc tests. The values in the bar graphs represent the mean ± SE. *p < 0.05, **p < 0.01, NS; not significant

**Fig. 5:**
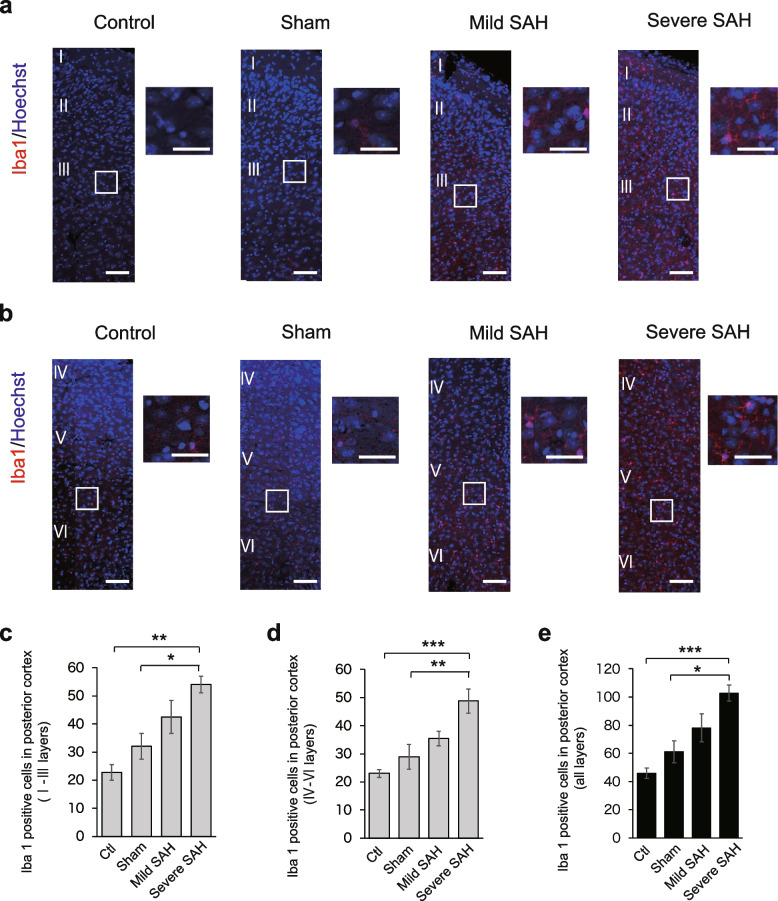
Iba1-positive microglia infiltrated layers I to VI of the cerebral cortex (dorsal region) in the posterior cerebral cortex (2.5 mm posterior to bregma). **a** Representative immunostaining images of Iba1-positive cells (red) in layers I to III of the posterior cerebrum in the control, sham, mild SAH, and severe SAH groups. Scale bar, 100 μm for the general figure and 50 μm for the enlarged figure. **b** Representative immunostaining images of Iba1-positive cells (red) in layers IV to VI of the posterior cerebrum in the control, sham, mild SAH, and severe SAH groups. The scale bar is 100 μm for the general figure and 50 μm for the enlarged figure. **c** Quantification of the number of Iba1-positive cells in layers I to III of the posterior region of the cerebrum (*n* = 4, each group, control vs. severe SAH; *p* = 0.001, sham vs. severe SAH; *p* = 0.028). **d** Quantification of the number of Iba1-positive cells in layers IV to VI of the posterior region of the cerebrum (*n* = 4, each group, control vs. severe SAH; *p* < 0.001, sham vs. severe SAH; *p* = 0.007). **e** Quantification of the number of Iba1-positive cells in all layers of the posterior region of the cerebrum (*n* = 4, each group, control vs. severe SAH; *p* < 0.001, sham vs. severe SAH; *p* = 0.010). Statistical analyses were performed with one-way ANOVA and Tukey–Kramer post hoc tests. The values in the bar graphs represent the mean±SE. *p < 0.05, **p < 0.01, ***p < 0.001

**Fig. 6:**
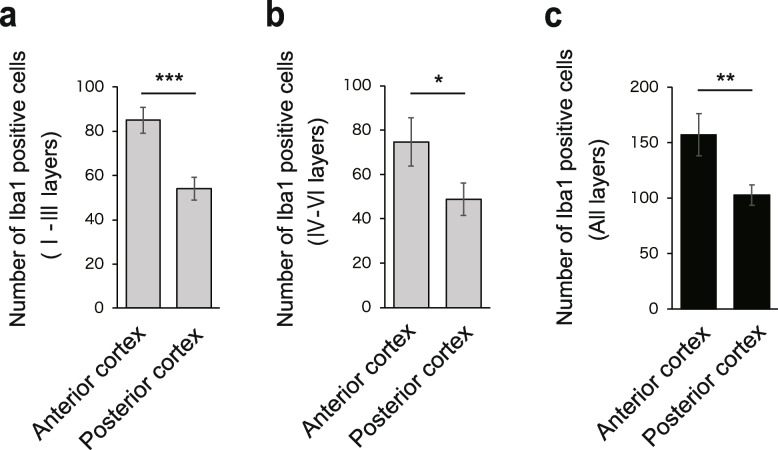
Inflammation is still induced away from the site of blood injection, but to a lesser degree. Iba1-positive cells increased away from the blood injection site, but to a lesser extent. I–III layers (**a**), IV–VI layers (**b**), and all layers (**c**). Statistical analyses were performed with an unpaired two-tailed Student's t test (two-sided). The values in the bar graphs represent the mean ± SD. *p < 0.05, **p < 0.01, ***p < 0.001

Next, we performed a similar analysis of the cortex in the intermediate (1.0 mm posterior to bregma) and posterior (2.5 mm posterior to bregma) regions of the cerebrum. In layers I to III (superficial layers) of the intermediate cortex, the number of Iba1-positive microglia increased with the severity of SAH, as in the anterior region (Fig. 4a, c). In layers IV to VI, the number of Iba1-positive microglia increased with the severity of SAH (Fig. 4b, d), and the number of active Iba1-positive microglia increased in all layers (Fig. 4e). The same result was observed in the posterior cortex (Fig. 5a–e). However, although the number of Iba1-positive activated microglia increased in the posterior part of the cerebrum after SAH injury, the number seemed to be slightly lower than that in the anterior region. In addition, in severe SAH, the anterior cortex had significantly more Iba1-positive cells than the posterior cortex, all in the superficial (Fig. 6a), deep (Fig. 6b), and all layers (Fig. 6c).

**Fig. 7:**
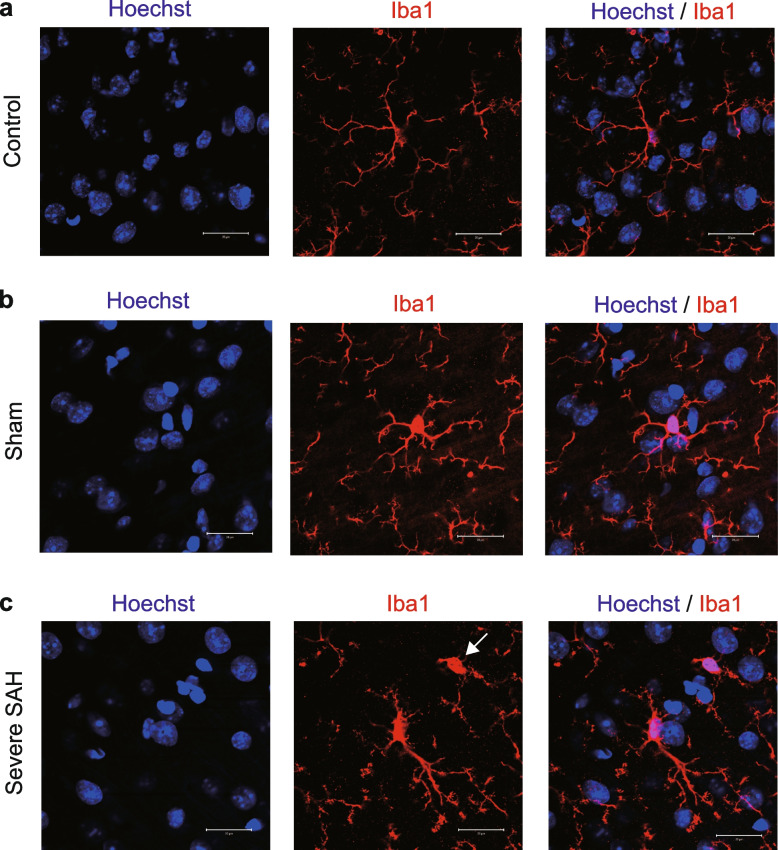
Microglia are morphologically transformed by SAH. **a** Immunostaining images of nuclear (blue) and Iba1 (red) staining in the control group; Iba1-positive cells have a ramified shape. **b** Immunostaining images of nuclear (blue) and Iba1 (red) staining in the sham group; Iba1-positive cells have a ramified shape. **c** Immunostaining images of nuclear (blue) and Iba1 (red) staining in SAH. Iba1-positive cells are hyperramified or reactive in shape. Some cells were amoeboids, as shown by the white arrows. Scale bars: 20 μm

Microglia are known to undergo morphological changes upon stimulation. They change their shape due to inflammation, transforming from a ramified structure with small cell bodies and long processes to a hyperramified or reactive structure with large cell bodies and thick, short processes. They also become amoeboid with phagocytic ability [25]. The Iba1-positive microglia observed in the present study were mainly ramified in the control and sham groups, whereas they were mainly hyperramified or reactive in the SAH group (Fig. 7a–c). A few Iba1-positive amoeboid-type cells were also observed (Fig. 7c). The results were similar regardless of depth in the cortical layers and showed that microglia undergo morphological remodeling under inflammatory conditions induced by SAH.

Reactive astrocytes are also known to be activated after traumatic brain injury (TBI) or ischemic stroke [26, 27] by activated microglia, which divide, proliferate, and enlarge to extend their projections [28]. We attempted to detect reactive astrocytes by GFAP immunostaining and the intraperitoneal administration of 5-*ethynyl*-2′-*deoxyuridine* (EdU) (50 mg/kg, i.p.) 1 hour before the dissection of the mice, but no reactive astrocytes were found in the cortices of mice in the control, sham, or any SAH groups (data not shown). The activation of astrocyte is reported to increase at approximately 3 days after brain injuries such as TBI [27], and similarly, reactive astrocytes were potentially observed a few days after SAH injury in the present study on SAH model mice.

### Neuronal cell death is increased in layers I to III of the cerebral cortex in correlation with neuroinflammation

We showed that in SAH model mice, activated microglia were induced from the anterior to posterior regions of the cerebrum and in all layers of the cerebral cortex depending on the severity of the injury. Since neuroinflammation triggers neuronal cell death in SAH and brain injury models [29–32], we investigated whether the worsening of neuroinflammation in all layers of the cortex led to neuronal cell death.

**Fig. 8:**
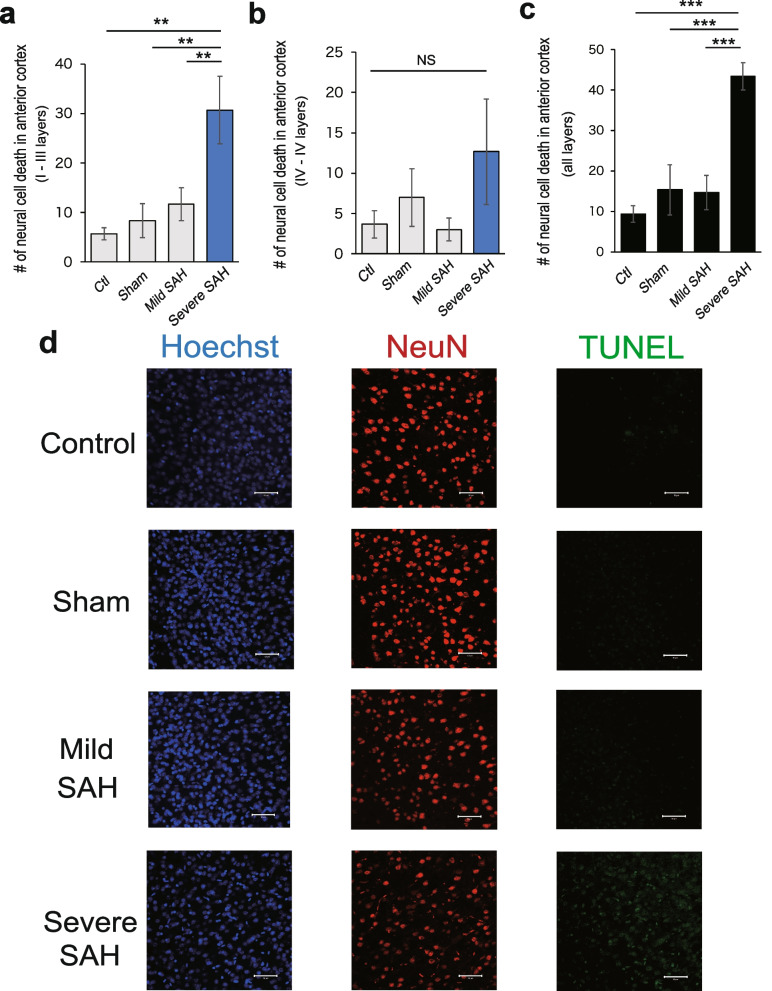
As the severity of SAH worsens, neuronal cell death increases in the cerebral cortex in the anterior region of the cerebrum (1.0 mm anterior to bregma). **a** Cells that were positive for both NeuN and TUNEL were quantified to assess neuronal cell death. The number of dead neural cells in layers I to III of the anterior region of the cerebrum was quantified (*n* = 3, each group). **b** Quantification of the number of dead neural cells in layers IV to VI of the anterior region of the cerebrum (*n* = 3, each group). **c** Quantification of the number of dead neural cells in all layers of the anterior region of the cerebrum (*n* = 3, each group). **d** Representative confocal images of TUNEL (green) and NeuN (red) in the anterior cortex. Nuclei were counterstained with Hoechst (blue). Scale bar, 50 μm. Statistical analyses were performed with one-way ANOVA and Tukey–Kramer post hoc tests. The values in the bar graphs represent the mean ± SD. **p < 0.01, ***p < 0.001. NS: not significant

**Fig. 9:**
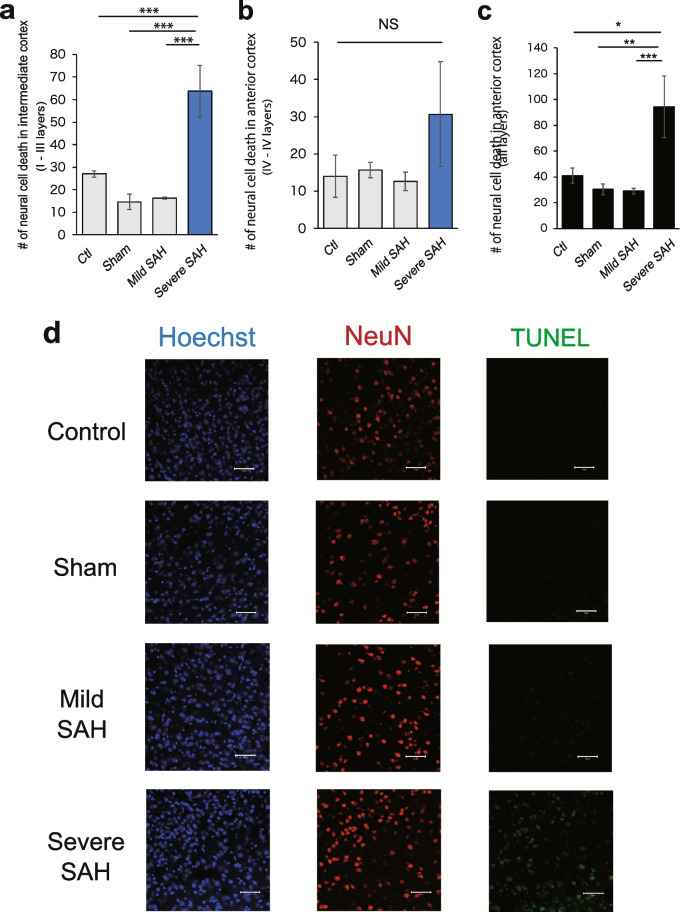
Neural cell death in the intermediate cerebral cortex (1.0 mm posterior to bregma). **a** Quantification of the number of dead neural cells in layers I to III of the intermediate region of the cerebrum (*n* = 3). **b** Quantification of the number of dead neural cells in layers IV to VI of the intermediate region of the cerebrum (*n* = 3, each group). **c** Quantification of the number of dead neural cells in all layers of the intermediate region of the cerebrum (*n* = 4, each group). **d** Representative confocal images of TUNEL (green) and NeuN (red) in the intermediate cortex. Nuclei were counterstained with Hoechst (blue). Scale bar, 50 μm. Statistical analyses were performed with one-way ANOVA and Tukey–Kramer post hoc tests. The values in the bar graphs represent the mean ± SD. *p < 0.05. **p < 0.01, ***p < 0.001. NS: not significant

**Fig. 10:**
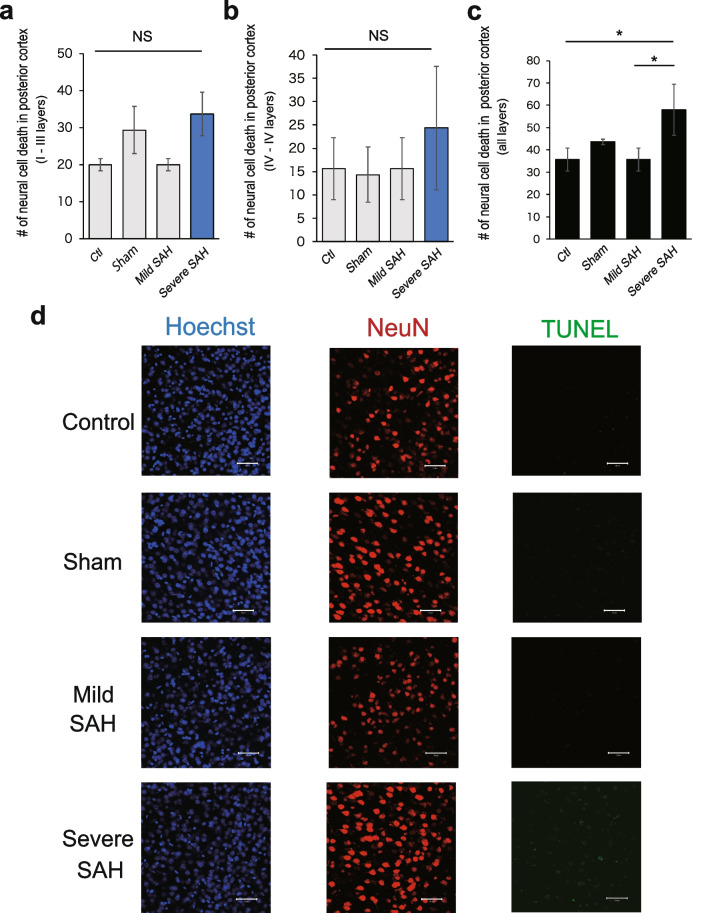
Neural cell death in the posterior cerebral cortex (2.5 mm posterior to bregma). **a** Cells that were positive for both NeuN and TUNEL were quantified to assess neuronal cell death. The number of dead neural cells in layers I to III of the posterior region of the cerebrum was quantified (*n* = 3, each group). **b** Quantification of the number of dead neural cells in layers IV to VI of the posterior region of the cerebrum (*n* = 3, each group). **c** Quantification of the number of dead neural cells in all layers of the posterior region of the cerebrum (*n* = 3, each group). **d** Representative confocal images of TUNEL (green) and NeuN (red) in the posterior cortex. Nuclei were counterstained with Hoechst (blue). Scale bar, 50 μm. Statistical analyses were performed with one-way ANOVA and Tukey–Kramer post hoc tests. The values in the bar graphs represent the mean ± SD. *p < 0.05, NS: not significant

We quantified the degree of cell death by immunostaining for neural NeuN, a marker nuclei in neurons [33], and terminal deoxynucleotidyl transferase dUTP nick end labeling (TUNEL) [34]. In the anterior part of the cerebrum, neuronal cell death increased with the severity of SAH in superficial layers I to III (Fig. 8a, d). In the deeper layers (IV to VI), neuronal cell death also tended to increase according to the severity of SAH, but not significant (Fig. 8b), and this phenomenon was observed in all layers of the cerebral cortex (Fig. 8c). In the intermediate of the cerebrum, similar to the anterior region, neuronal cell death increased as the severity of SAH worsened (Figs. 9a–d). And the difference was not significant in the posterior cortex (Fig. 10a–d).

### Neuroinflammation in the hippocampus is milder than that in the cerebral cortex

**Fig. 11:**
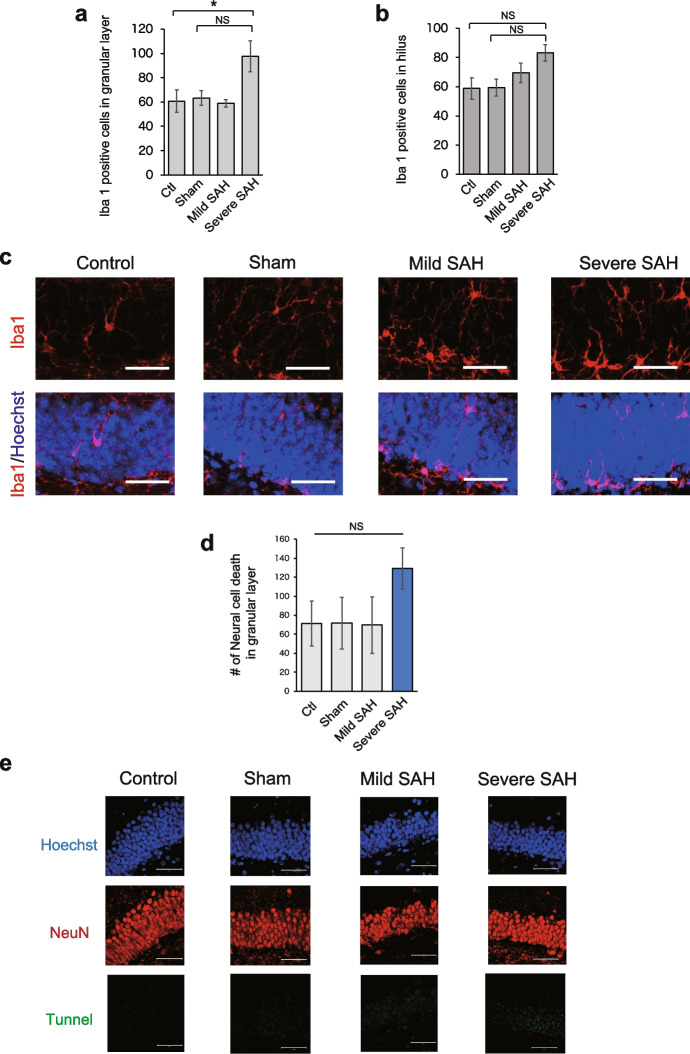
Neuroinflammation and neural cell death in the hippocampus. **a** Quantification of the number of Iba1-positive cells in the granular layer (*n* = 4, each group, control vs. severe SAH; *p* = 0.043, sham vs. severe SAH; *p* = 0.062). **b** Quantification of the number of Iba1-positive cells in the hilus (*n* = 4, each group, control vs. severe SAH; *p* = 0.085, sham vs. severe SAH; *p* = 0.097). **c** Representative immunostaining images of Iba1-positive cells (red) in the hippocampi of mice in the control, sham, mild SAH, and severe SAH groups. Scale bar, 100 μm. **d** Quantification of the number of dead neural cells in the granular layer (*n* = 3, each group. **e** Representative confocal images of TUNEL (green) and NeuN (red) in the hippocampus. Scale bar, 100 μm. Statistical analyses were performed with one-way ANOVA and Tukey–Kramer post hoc tests. The values in the bar graphs represent the mean ± SE. NS: not significant

Next, we analyzed the degree of neuroinflammation by quantifying the number of Iba1-positive microglia in the hippocampus, which is located deeper from the pia mater than the cerebral cortex. Since SAH has been reported to damage the dentate gyrus of the hippocampus due to ischemia [35, 36], we focused on this region to investigate how neuroinflammatory damage extends beyond the cerebral cortex. We quantified the number of Iba1-positive microglia in the granular cell layer of the hippocampus in each group and found that the number was significantly increased in the severe SAH model mice (Fig. 11a, c and Supplementary Figure S1a). The number of microglia in the hilus was also slightly increased in the severe SAH model (Fig. 11b and Supplementary Figure S1a). However, the number of induced microglia was lower than in the cortex, and the number of Iba1-positive microglia was not significantly increased in hilus, although there was an increasing trend.

The extent of neuronal cell death was also quantified using TUNEL staining and NeuN. In the granular cell layer, neuronal cell death tended to increase in the mild SAH model but not significant (Fig. 11d, e).

The hippocampus is well known to be vulnerable to SAH and susceptible to damage [11, 37, 38], and SAH-induced ischemia has been reported to be responsible for these phenotypes [36, 39, 40]. In the granular cell layer and hilus of the hippocampal dentate gyrus, the effects of neuroinflammation may be limited. This suggests that other factors, such as ischemia, are responsible for neuronal cell death.

### Effect of neuroinflammation on adult neurogenesis

The above analysis showed that the degree of neuroinflammation in the hippocampus, which is located deeper than the cortex, was milder than that in the cortex. We examined whether SAH-induced neuroinflammation in the hippocampus, which is located deeper than the cortex, affects other functions.

One of the characteristic functions of the hippocampus and ventricular-subventricular zone (V-SVZ) is adult neurogenesis [41, 42]. In the hippocampus, neurogenesis occurs throughout life, allowing for new memories [43], and neurons generated in the V-SVZ migrate to the olfactory bulb, where they contribute to odor identification and sexual behavior [44]. This neurogenesis is known to be impaired by neuroinflammation [45].

Here, we investigated whether neuroinflammation caused by SAH also impairs adult neurogenesis. We used doublecortin (DCX) [46] as a marker for newly generated neurons. In the V-SVZ, the number of DCX-positive cells did not differ between the severe SAH, control and sham groups but was significantly increased in the mild SAH group (Supplementary Figure S2a and b). In the hippocampus, the number of DCX-positive cells did not differ among the control, sham, and severe SAH groups, but was mildly increased in the mild SAH group (Supplementary Figure S2c and d). The increase in the number of DCX-positive cells in the mild SAH group was unexpected, suggesting that mild inflammation is a convenient stimulus for DCX upregulation. However, in this study protocol, brain sections were analyzed 1 day after SAH injury, and it would thus be reasonable to assume that DCX expression was increased by inflammatory stimuli rather than that neurogenesis was increased. To determine whether neurogenesis is sustained, mid- to long-term analyses after SAH injury are needed.

## Discussion

In this study, we found that neuroinflammation spilled over from superficial to deep layers in the cerebral cortices of mice after SAH injury, leading to neuronal cell death, especially in the anterior cortex. Neuroinflammation and neuronal cell death increased with the severity of SAH.

In clinical practice, SAH patients present with a variety of clinical manifestations, ranging from mild disease with few sequelae to severe disease with severe sequelae or even death [37, 38]. Therefore, to study the pathogenesis of the disease, it was necessary to analyze the disease according to its severity and therefore created model mice with mild and severe disease. There are two main experimental models of SAH, the injection model [37, 47] in which hematoma is injected into the prechiasmatic cistern or cisterna magna, and the perforation model [48, 49], in which the tip of the internal carotid artery is punctured with nylon thread or wire. The perforation model is probably the most popular model in recent experimental SAH studies but is limited by the fact that the amount of blood spurting and intracranial pressure cannot be controlled, resulting in inconsistent brain damage due to SAH [50]. On the other hand, the intracranial pressure does not increase substantially in the injection model [51]. As such, we herein made it possible to adjust the amount and time of hematoma injection and ensured the results by simultaneously measuring the intracranial pressure. We tried several combinations of the blood volume and injection time and decided to target the elevation of intracranial pressure at approximately the same level as the elevated blood pressure during SAH, which is considered equivalent to the global ischemia status of intracranial blood flow [52], for the creation of the severe disease model. Furthermore, mice with lower neurological scores at 24 h were included in the analysis of the severe SAH model, and those with scores that were too high excluded. For the mild SAH model, we created a model in which the intracranial pressure was elevated but the neurological scores were not too low.

In the acute phase of SAH, microglia plays exclusive role to neuronal apoptosis [53]. Also, residual microglia activate 24 h after SAH and macrophage does not appear until 72 h later [54]. Within 24 h of SAH, the residual microglia show morphological changes in their activation [24], reaching a peak, which is more common in brain regions adjacent to the hematoma [55]. Some reports suggest that SAH may cause damage in remote areas; the expression of inflammatory cytokines is upregulated in the cerebral cortex adjacent to and distal to the hematoma in experimental SAH [56]. Additionally, microglia are upregulated in not only the vicinity of the hematoma but also the motor cortex following SAH [24]. Despite this, how far the inflammatory changes associated with microglial invasion after SAH extend into the cerebrum remained unknown.

In our study, we showed that neuroinflammation was increased in all layers of the cerebral cortex contralateral to the basal cistern where the hematoma was injected. Furthermore, this result was similar in not only the anterior region of the cerebrum but also the intermediate and posterior regions. This result suggests that the inflammatory changes associated with SAH are widespread throughout the cerebrum. With respect to inflammation after SAH, the effects of heme and other degradation products of blood are known [57]. However, in remote areas, hematomas are thought to have less direct effects. Therefore, the activation of neuroinflammation in remote areas may not be achieved only by interaction with heme and microglia but rather may include other insults, such as transient global ischemia, ischemia due to acute vasospasm, and a dysregulated inflammatory response after stroke [58–60]. In addition, the interaction between microglia and neurons contributes to neuronal apoptosis after SAH, and depletion of microglia significantly inhibits neuronal apoptosis in experimental SAH [31, 53]. We also showed that neuronal cell death was widespread in the cerebrum and that it was correlated with the extent of neuroinflammation. These results indicate that SAH-induced neuroinflammation extends to the entire cerebrum and causes neuronal cell death.

In our results, the degree of neuroinflammation was lower in the granular layer and hilus of the hippocampus, which is located deeper than the cerebral cortex, than in the cerebral cortex. This result may suggest that a mechanism other than inflammation is responsible for neuronal cell death in the hippocampus. In general, the hippocampus is vulnerable to ischemia [61], and whole-brain ischemia caused by SAH are thought to affect the hippocampus [36]. Therefore, ischemia and/or other effects, but not inflammation, were thought to responsible for the hippocampal damage caused by SAH.

Since the cerebral cortex controls movement [62] and long-term memory [63] and since neuroinflammation can impair motor function and long-term memory retention even when intracranial hypertension [64] or physical damage [65] caused by SAH is mild, reducing inflammation throughout the cerebrum may be a promising therapeutic strategy. Although some studies using mouse models of SAH have reported that anti-inflammatory drugs have therapeutic effects [66, 67], there is still no clear evidence that anti-inflammatory treatment strategies can be useful for patients with SAH. Previous studies have not shown that the therapeutic effect is due to the suppression of neuroinflammation spreading throughout the cerebrum. Our results suggest that the therapeutic target for inflammatory changes in the acute phase of SAH is not localized but extends to the entire brain. Thus, in the future, preclinical studies should be conducted to determine which anti-inflammatory drugs should be administered and when and how they should be administered to achieve the best therapeutic effects.

### Comparison with previous reports

The central theme of the study by Zheng et al. [24] was microglial activation and polarity. This study showed that SAH increased the number of microglia near the hematoma, in the motor cortex, and the hippocampus. In contrast, our study is novel in that it showed that inflammation was induced in the entire cerebral cortex. In terms of hematoma in a remote area, our study was more detailed, showing that even in the remote area, from the anterior to the posterior cortex, respectively, and superficial to deep layers, respectively, inflammation was induced. Therefore, we believe that our results show considerably more findings than this study in terms of how brain damage due to SAH extends throughout the cerebrum and how the damage depends on the severity of the injury.

The previous study by Duris et al. [56] showed that inflammatory cytokines are elevated in the distal cortex of the hematoma after SAH. Again, we have newly shown from our result that there is an associated increase in neuronal death, which is severity-dependent.

Sabri et al. [37] showed that SAH causes microvessel thrombosis and increased cell death in a specific brain area. Although it is the same in the sense that we are studying cell death caused by the acute phase of SAH, this is substantially different from our research about neuroinflammation and neuronal cell death in the entire cortex.

Overall, compared to previous studies showing acute remote inflammation, this study newly shows that EBI causes neuroinflammation and neuronal cell death throughout the brain and that its severity is consistent with the magnitude of the load on the animal model.

### Limitations of this study

First, although we revealed the increase of the number of Iba1-positive cells after SAH, this study does not confirm whether these phenomena are the cause or result of tissue injury. Additional experiments under inhibition of microglial activation will be needed to address the cause-and-effect relationship between microglia-mediated neuroinflammation and neuronal cell death.

Second, although Iba1 was used as a marker of neuroinflammation in this study, strictly speaking, Iba1 cannot distinguish between microglia and macrophages. Therefore, it is necessary to distinguish between microglia and macrophages by checking the expression of other specific markers.

## Conclusion

Analysis of the SAH mouse model revealed that EBI-induced inflammation and neuronal cell death were not localized but rather extended to the entire cerebrum, with inflammatory damage generally occurring in all layers of the cerebral cortex. Furthermore, the degree of neuroinflammation increased as the severity of the disease increased, causing neuronal cell death. As a future prospect, suppression of inflammation in the entire cerebrum is suggested to be a useful therapeutic strategy for EBI.

## Methods

### Mice

All experimental procedures were approved by the ethics committee of Keio University and performed in accordance with the Guide for the Care and Use of Laboratory Animals (U.S. National Institutes of Health). Male C57BL/6J mice (12–14 weeks old) were purchased from Sankyo Lab Service (Tokyo, Japan).

### SAH induction

In creating a mouse model of subarachnoid hemorrhage, we implemented the anterior injection method [68]. Although several methods have been reported for creating SAH models [46, 47], no perfect model is available. Since our goal was to create both a mild and a severe model, we adopted a method that allowed us to intentionally adjust the severity of the disease by ourselves depending on the amount and speed of the hematoma injection. Mice were anesthetized by the intraperitoneal injection (0.01 mL/g) of a mixture of midazolam (4 mg/kg), dexmedetomidine hydrochloride (0.75 mg/kg), and butorphanol tartrate (5 mg/kg), and the procedure was performed under spontaneous breathing; the mouse body temperature was maintained at 37.0 °C ± 0.5 °C using a thermometer and a heating pad. The head of the mouse was fixed with a stereotaxic instrument (David Kopf Instruments, Los Angeles, USA), and a midline scalp incision was made from anterior to the bregma to the occipital bone. A burr hole was drilled 4.5 mm anterior to bregma and 1 mm lateral to the right side. A 27-G spinal needle was inserted 45° caudally until it hit the skull base (typically approximately 7 mm deep) and was then pulled back 0.5 mm to reach the subarachnoid space. A burr hole was also drilled on the contralateral side, and a fiber-optic pressure transducer and 0.9 Fr tip catheter (Neuroscience incorporation, Tokyo, Japan) was inserted to measure the intracranial pressure. During the intracranial pressure measurement, nonheparinized blood obtained from the left atrium of the homogeneous isochronic mouse was injected using a stereotaxic injector (Muromachi Kikai Corporation, Tokyo, Japan). In the mild disease model, 40 μl of blood was injected over 45 s, whereas 80 μl of blood was injected over 45 s in the severe disease model. After the injection, the needle was kept in the same position for 3 min to prevent backflow of blood and was then removed. Sham mice were only subjected to needle insertion and were not injected with blood. Control mice received only anesthesia and no needle insertion.

### Neurological scoring

A modified Garcia score [69, 70] with a score of 18 points was evaluated to assess neurological function at two time points: just prior to the induction of SAH and just prior to sacrifice 24 h later. Mice with a score of 16 to 18 points after 24 h were adopted as mild disease model mice, and those with a score of 16 points or less were adopted as severe disease model mice.

### Immunostaining

For immunohistochemistry, vibratome sections were prepared by standard protocols after perfusion and fixation with 4% PFA/PBS. The sections were treated with 3% H2O2/PBS for 15 min to quench endogenous peroxidase activity and then processed for the retrieval of antigens optimized for each antibody as described below. Subsequently, the sections were permeabilized with 0.3% Triton X-100 (Sigma–Aldrich, X100-1GA)/PBS for 30 min at room temperature. After blocking with blocking One buffer (Nacalai Tesque, 03953-95) for 30 min at room temperature, the sections were incubated at 4 °C overnight with the following antibodies: rabbit monoclonal anti-Iba1 (Fujifilm Wako Chemicals, 019-19741; 1:500), mouse monoclonal anti-NeuN (Millipore, MAB377; 1:500), (Cell Signaling Technology, 9661; 1:500), rat monoclonal anti-GFAP (Invitrogen, 13-0300; 1:500), and goat polyclonal anti-DCX (Santa Cruz Biotechnology, sc-8066; 1:500). After washing with PBS three times, the sections were incubated for 90 min at room temperature with secondary antibodies conjugated to biotinylated substrates (Jackson ImmunoResearch, 111-065-144 or 712-065-153 or 115-065-146; 1:500). The signals were then enhanced with a VECTASTAIN Elite ABC HRP Kit (Vector Laboratories, PK-6100) and analyzed with a TSA™ Fluorescein System (Perkin Elmer, NEL701001KT or NEL702001KT). To detect cell death, we used In Situ Cell Death Detection Kit, Fluorescein (Roche, 11684795910) in accordance with the manufacturer’s instructions. Cell nuclei of the sections were counterstained with Hoechst 33258 (Sigma–Aldrich, B2883; 10 μg/mL). The sections were mounted on glass slides and analyzed with a confocal laser scanning microscope LSM700 (Carl Zeiss). Tiling images of the cortex and hippocampus of the samples were taken using a × 40 objective lens, and the number of Iba1-positive cells and NeuN-TUNEL (sigma-aldrich/Roche, In Situ Cell Death Detection Kit, Fluorescein; Cat. 11684795910)-positive cells were manually calculated.

### Statistical analysis

For each statistical analysis, at least three independent experiments were conducted. Statistical significance was determined by the unpaired two-tailed Student’s *t* test (two-tailed) and one-way analysis of variance followed by the Tukey–Kramer post hoc test for the comparison of two data sets. Throughout the study, *p values* of less than 0.05 were considered statistically significant. The following significance thresholds were used throughout: *p < 0.05, **p < 0.01, ***p < 0.001; NS, not significant. The values in bars and line graphs represent the mean ± SE for the rest of the figures.

### RNA-seq data analysis

Original data were downloaded from Gene Expression Omnibus and the RKPM values were transformed into log_2_(RKPM+1). The expression matrix of microglial markers (*Aif1*, *Cx3cr1*, *Cd68*, *P2ry12*, *P2ry13*, *Tmem119, Gpr34*, *Siglech*, and *Trem2*) was transformed by principal component analysis. The correlation coefficients among those genes were also calculated.

## Supplementary Information

**Additional file 1.** Supplementary Figure S1, Neuroinflammation in the hippocampus. (a) Confocal images of merged Iba 1 (red) and Hoechst (blue) in the overall view of the hippocampus in control, sham, mild SAH, and severe SAH mice. Scale bar, 100 μm. Supplementary Figure S2, Adult neurogenesis in the V-SVZ and SGZ. (a) Quantification of the number of DCX-positive cells in the V-SVZ (n=4, each group, control vs. mild SAH; p=0.022). (b) Representative confocal images of DCX (red) in the V-SVZ. Scale bar, 100 μm. (c) Quantification of the number of DCX-positive cells in the SGZ (n=4, each group, control vs. mild SAH; p=0.268). (d) Representative confocal images of DCX (red) in the SGZ. Scale bar, 100 μm. Statistical analyses were performed with one-way ANOVA and Tukey–Kramer post hoc tests. The values in the bar graphs represent the mean±SE. *p<0.05, NS; not significant.

## Data Availability

The datasets generated during and/or analyzed during the current study are available from the corresponding author on reasonable request.

## Abbreviations

EBI: Early brain injury
RNA-seq: RNA sequencing
SAH: Subarachnoid hemorrhage
SGZ: Subgranular zone
V-SVZ: Ventricular-subventricular zone

## References

[1] Macdonald RL, Schweizer TA (2017). Spontaneous subarachnoid haemorrhage. Lancet..

[2] Neifert SN, Chapman EK, Martini ML, Shuman WH, Schupper AJ, Oermann EK, Mocco J, Macdonald RL (2021). Aneurysmal subarachnoid hemorrhage: the last decade. Transl Stroke Res.

[3] Nieuwkamp DJ, Setz LE, Algra A, Linn FHH, de Rooij NK, Rinkel GJE (2009). Changes in case fatality of aneurysmal subarachnoid haemorrhage over time, according to age, sex, and region: a meta-analysis. Lancet Neurol.

[4] Etminan N, Chang HS, Hackenberg K, de Rooij NK, Vergouwen MDI, Rinkel GJE, Algra A (2019). Worldwide incidence of aneurysmal subarachnoid hemorrhage according to region, time period, blood pressure, and smoking prevalence in the population. JAMA Neurol.

[5] van Gijn J, Kerr RS, Rinkel GJE (2007). Subarachnoid haemorrhage. Lancet..

[6] Gonçalves B, Turon R, Mendes A, Melo N, Lacerda P, Brasil P, Bozza FA, Kurtz P, Righy C (2018). Effect of early brain infarction after subarachnoid hemorrhage: a systematic review and meta-analysis. World Neurosurg.

[7] Caner B, Hou J, Altay O, Fuj M, Zhang JH (2012). Transition of research focus from vasospasm to early brain injury after subarachnoid hemorrhage. J Neurochem.

[8] Fujii M, Yan J, Rolland WB, Soejima Y, Caner B, Zhang JH (2013). Early brain injury, an evolving frontier in subarachnoid hemorrhage research. Transl Stroke Res.

[9] Chou SHY, Feske SK, Simmons SL, Konigsberg RGJ, Orzell SC, Marckmann A, Bourget G, Bauer DJ, De Jager PL, Du R, Arai K, Lo EH, Ning MM (2011). Elevated peripheral neutrophils and matrix metalloproteinase 9 as biomarkers of functional outcome following subarachnoid hemorrhage. Transl Stroke Res.

[10] Li Y, Wu P, Bihl JC, Shi H (2020). Underlying mechanisms and potential therapeutic molecular targets in blood-brain barrier disruption after subarachnoid hemorrhage. Curr Neuropharmacol.

[11] Han SM, Wan H, Kudo G, Foltz WD, Vines DC, Green DE, Zoerle T, Tariq A, Brathwaite S, D'Abbondanza J, Ai J, Macdonald RL (2014). Molecular alterations in the hippocampus after experimental subarachnoid hemorrhage. J Cereb Blood Flow Metab.

[12] Sehba FA, Pluta RM, Zhang JH (2011). Metamorphosis of subarachnoid hemorrhage research: from delayed vasospasm to early brain injury. Mol Neurobiol.

[13] Tso MK, Turgeon P, Bosche B, Lee CK, Nie T, D’Abbondanza J, Ai J, Marsden PA, Macdonald RL (2021). Gene expression profiling of brain endothelial cells after experimental subarachnoid haemorrhage. Sci Rep.

[14] Geraghty JR, Davis JL, Testai FD (2019). Neuroinflammation and microvascular dysfunction after experimental subarachnoid hemorrhage: emerging components of early brain injury related to outcome. Neurocrit Care.

[15] Sun CM, Enkhjargal B, Reis C, Zhou KR, Xie ZY, Wu LY, Zhang TY, Zhu QQ, Tang JP, Jiang XD, Zhang JH (2019). Osteopontin attenuates early brain injury through regulating autophagy-apoptosis interaction after subarachnoid hemorrhage in rats. CNS Neurosci Ther.

[16] Pan P, Xu L, Zhang H, Liu Y, Lu X, Chen G, Tang H, Wu J (2020). A review of hematoma components clearance mechanism after subarachnoid hemorrhage. Front Neurosci.

[17] Ma B, Day JP, Phillips H, Slootsky B, Tolosano E, Doré S (2016). Deletion of the hemopexin or heme oxygenase-2 gene aggravates brain injury following stroma-free hemoglobin-induced intracerebral hemorrhage. J Neuroinflammation.

[18] Wong GC, Chen J, Zheng Z, Lu G, Chan W, Zhang Y (2022). Microglia activation, classification and microglia-mediated neuroinflammatory modulators in subarachnoid hemorrhage. Neural Regen Res.

[19] Zeyu Z, Yuanjian F, Cameron L, Sheng C (2021). The role of immune inflammation in aneurysmal subarachnoid hemorrhage. Exp Neurol.

[20] Zheng VZ, Wong GKC (2017). Neuroinflammation responses after subarachnoid hemorrhage: a review. J Clin Neurosci.

[21] Peng J, Wu Y, Tian X, Pang J, Kuai L, Cao F, Qin X, Zhong J, Li X, Li Y, Sun X, Chen L, Jiang Y (2017). High-throughput sequencing and co-expression network analysis of lncRNAs and mRNAs in early brain injury following experimental subarachnoid haemorrhage. Sci Rep.

[22] Ito D, Imai Y, Ohsawa K, Nakajima K, Fukuuchi Y, Kohsaka S (1998). Microglia-specific localisation of a novel calcium binding protein, Iba1. Brain Res Mol Brain Res.

[23] Wen YR, Tan PH, Cheng JK, Liu YC, Ji RR (2011). Microglia: a promising target for treating neuropathic and postoperative pain, and morphine tolerance. J Formos Med Assoc.

[24] Zheng ZV, Lyu H, Lam SYE, Lam PK, Poon WS, Wong GKC (2020). The dynamics of microglial polarization reveal the resident neuroinflammatory responses after subarachnoid hemorrhage. Transl Stroke Res.

[25] Hinwood M, Morandini J, Day TA, Walker FR (2012). Evidence that microglia mediate the neurobiological effects of chronic psychological stress on the medial prefrontal cortex. Cereb Cortex.

[26] Morizawa YM, Hirayama Y, Ohno N, Shibata S, Shigetomi E, Sui Y, Nabekura J, Sato K, Okajima F, Takebayashi H, Okano H, Koizumi S (2017). Reactive astrocytes function as phagocytes after brain ischemia via ABCA1-mediated pathway. Nat Commun.

[27] Shinozaki Y, Shibata K, Yoshida K, Shigetomi E, Gachet C, Ikenaka K, Tanaka KF, Koizumi S (2017). Transformation of astrocytes to a neuroprotective phenotype by microglia via P2Y 1 receptor downregulation. Cell Rep.

[28] Pekny M, Pekna M (2014). Astrocyte reactivity and reactive astrogliosis: costs and benefits. Physiol Rev.

[29] Wu Y, Pang J, Peng J, Cao F, Guo Z, Jiang L, Teng Z, Huang Z, Cheng C, Jiang Y, Sun X (2019). Apolipoprotein E deficiency aggravates neuronal injury by enhancing neuroinflammation via the JNK/c-jun pathway in the early phase of experimental subarachnoid hemorrhage in mice. Oxid Med Cell Longev.

[30] Yuan B, Zhou X, You Z, Xu W, Fan J, Chen S, Han Y, Wu Q, Zhang X (2020). Inhibition of AIM2 inflammasome activation alleviates GSDMD-induced pyroptosis in early brain injury after subarachnoid haemorrhage. Cell Death Dis.

[31] Lu Y, Zhang XS, Zhang ZH, Zhou XM, Gao YY, Liu GJ, Wang H, Wu LY, Li W, Hang CH (2018). Peroxiredoxin 2 activates microglia by interacting with toll-like receptor 4 after subarachnoid hemorrhage. J Neuroinflammation.

[32] Zhang XS, Wu Q, Wu LY, Ye ZN, Jiang TW, Li W, Zhuang Z, Zhou ML, Zhang X, Hang CH (2016). Sirtuin 1 activation protects against early brain injury after experimental subarachnoid hemorrhage in rats. Cell Death Dis.

[33] Lind D, Franken S, Kappler J, Jankowski J, Schilling K (2005). Characterization of the neuronal marker NeuN as a multiply phosphorylated antigen with discrete subcellular localization. J Neurosci Res.

[34] Negoescu A, Lorimier P, Labat-Moleur F (1996). In situ apoptotic cell labeling by the TUNEL method: improvement and evaluation on cell preparations. J Histochem Cytochem.

[35] Veldeman M, Coburn M, Rossaint R, Clusmann H, Nolte K, Kremer B, Höllig A (2017). Xenon reduces neuronal hippocampal damage and alters the pattern of microglial activation after experimental subarachnoid hemorrhage: a randomized controlled animal trial. Front Neurol.

[36] Makino K, Osuka K, Watanabe Y, Usuda N, Hara M, Aoyama M, Takayasu M, Wakabayashi T (2015). Increased ICP promotes CaMKII-mediated phosphorylation of neuronal NOS at Ser847 in the hippocampus immediately after subarachnoid hemorrhage. Brain Res.

[37] Sabri M, Ai J, Lakovic K, D’abbondanza J, Ilodigwe D, Macdonald RL (2012). Mechanisms of microthrombi formation after experimental subarachnoid hemorrhage. Neuroscience..

[38] Tariq A, Ai J, Chen G, Sabri M, Jeon H, Shang X, Macdonald RL (2010). Loss of long-term potentiation in the hippocampus after experimental subarachnoid hemorrhage in rats. Neuroscience..

[39] Wada K, Osuka K, Watanabe Y, Usuda N, Fukasawa M, Araki Y, Okamoto S, Wakabayashi T (2018). Subarachnoid hemorrhage induces neuronal nitric oxide synthase phosphorylation at Ser1412 in the dentate gyrus of the rat brain. Nitric Oxide.

[40] Yin B, Xu Y, Wei RL, He F, Luo B, Wang J (2015). Inhibition of receptor-interacting protein 3 upregulation and nuclear translocation involved in necrostatin-1 protection against hippocampal neuronal programmed necrosis induced by ischemia/reperfusion injury. Brain Res.

[41] Ming GL, Song H (2011). Adult neurogenesis in the mammalian brain: significant answers and significant questions. Neuron..

[42] Kase Y, Shimazaki T, Okano H (2020). Current understanding of adult neurogenesis in the mammalian brain: how does adult neurogenesis decrease with age?. Inflamm Regen.

[43] van Praag H, Schinder AF, Christie BR, Toni N, Palmer TD, Gage FH (2002). Functional neurogenesis in the adult hippocampus. Nature..

[44] Lim DA, Alvarez-Buylla A (2016). The adult ventricular–subventricular zone (V-SVZ) and olfactory bulb (OB) neurogenesis. Cold Spring Harb Perspect Biol.

[45] Kohman RA, Rhodes JS (2013). Neurogenesis, inflammation and behavior. Brain Behav Immun.

[46] Kase Y, Otsu K, Shimazaki T, Okano H (2019). Involvement of p38 in age-related decline in adult neurogenesis via modulation of Wnt signaling. Stem Cell Rep.

[47] Pedard M, El Amki M, Lefevre-Scelles A, Compère V, Castel H. Double direct injection of blood into the cisterna magna as a model of subarachnoid hemorrhage. J Vis Exp. 2020. 10.3791/61322. 10.3791/61322 32925881

[48] Kamii H, Tominaga T, Chen J, Xu ZC, Xu XM, Zhang JH (2009). Filament perforation subarachnoid hemorrhage: mouse model. Animal models of acute neurological injuries. Springer protocols handbooks.

[49] Muroi C, Fujioka M, Okuchi K, Fandino J, Keller E, Sakamoto Y, Mishima K, Iwasaki K, Fujiwara M (2014). Filament perforation model for mouse subarachnoid hemorrhage: surgical-technical considerations. Br J Neurosurg.

[50] Prunell GF, Mathiesen T, Diemer NH, Svendgaard NA (2003). Experimental subarachnoid hemorrhage: subarachnoid blood volume, mortality rate, neuronal death, cerebral blood flow, and perfusion pressure in three different rat models. Neurosurgery..

[51] Lee JY, Sagher O, Keep R, Hua Y, Xi G (2009). Comparison of experimental rat models of early brain injury after subarachnoid hemorrhage. Neurosurgery..

[52] Zoerle T, Lombardo A, Colombo A, Longhi L, Zanier ER, Rampini P, Stocchetti N (2015). Intracranial pressure after subarachnoid hemorrhage. Crit Care Med.

[53] Hanafy KA (2013). The role of microglia and the TLR4 pathway in neuronal apoptosis and vasospasm after subarachnoid hemorrhage. J Neuroinflammation.

[54] Zhen X (2019). Resident Microglia Activate before Peripheral Monocyte Infiltration and p75NTR Blockade Reduces Microglial Activation and Early Brain Injury after Subarachnoid Hemorrhage. ACS Chem Nerosci.

[55] Provencio JJ, Altay T, Smithason S, Moore SK, Ransohoff RM (2011). Depletion of Ly6G/C+ cells ameliorates delayed cerebral vasospasm in subarachnoid hemorrhage. J Neurochem.

[56] Duris K, Lipkova J, Splichal Z, Madaraszova T, Jurajda M (2018). Early inflammatory response in the brain and anesthesia recovery time evaluation after experimental subarachnoid hemorrhage. Transl Stroke Res.

[57] Blackburn SL, Kumar PT, McBride D, Zeineddine HA, Leclerc J, Choi HA, Dash PK, Grotta J, Aronowski J, Cardenas JC, Doré S (2018). Unique contribution of haptoglobin and haptoglobin genotype in aneurysmal subarachnoid hemorrhage. Front Physiol.

[58] Offner H, Hurn PD (2012). A novel hypothesis: regulatory B lymphocytes shape outcome from experimental stroke. Transl Stroke Res.

[59] Hasegawa Y, Suzuki H, Uekawa K, Kawano T, Kim-Mitsuyama S (2015). Characteristics of cerebrovascular injury in the hyperacute phase after induced severe subarachnoid hemorrhage. Transl Stroke Res.

[60] Liesz A, Kleinschnitz C (2016). Regulatory T cells in post-stroke immune homeostasis. Transl Stroke Res.

[61] Bartsch T, Wulff P (2015). The hippocampus in aging and disease: from plasticity to vulnerability. Neuroscience..

[62] Li N, Chen TW, Guo ZV, Gerfen CR, Svoboda K (2015). A motor cortex circuit for motor planning and movement. Nature..

[63] Ghazizadeh A, Hong S, Hikosaka O (2018). Prefrontal cortex represents long-term memory of object values for months. Curr Biol.

[64] Florez WA, García-Ballestas E, Deora H, Agrawal A, Martinez-Perez R, Galwankar S, Keni R, Menon GR, Joaquim A, Moscote-Salazar L-R (2021). Intracranial hypertension in patients with aneurysmal subarachnoid hemorrhage: a systematic review and meta-analysis. Neurosurg Rev.

[65] Lee GY, Ryu CW, Ko HC, Jahng GH (2020). Correlation between gray matter volume loss followed by aneurysmal subarachnoid hemorrhage and subarachnoid hemorrhage volume. Neuroradiology..

[66] Tosun C, Kurland DB, Mehta R, Castellani RJ, deJong JL, Kwon MS, Woo SK, Gerzanich V, Simard JM (2013). Inhibition of the Sur1-Trpm4 channel reduces neuroinflammation and cognitive impairment in subarachnoid hemorrhage. Stroke..

[67] Pradilla G, Thai QA, Legnani FG, Hsu W, Kretzer RM, Wang PP, Tamargo RJ (2004). Delayed intracranial delivery of a nitric oxide donor from a controlled-release polymer prevents experimental cerebral vasospasm in rabbits. Neurosurgery..

[68] Sabri M, Jeon H, Ai J, Tariq A, Shang X, Chen G, Macdonald RL (2009). Anterior circulation mouse model of subarachnoid hemorrhage. Brain Res.

[69] Garcia JH, Wagner S, Liu K-F, Hu XJ (1995). Neurological deficit and extent of neuronal necrosis attributable to middle cerebral artery occlusion in rats. Stroke..

[70] Sugawara T, Ayer R, Jadhav V, Zhang JH (2008). A new grading system evaluating bleeding scale in filament perforation subarachnoid hemorrhage rat model. J Neurosci Methods.

