# Supplementary figures and images for: Subarachnoid hemorrhage triggers neuroinflammation of the entire cerebral cortex, leading to neuronal cell death
Source: Inflamm Regen. 2022 Dec 14;42:61. doi: 10.1186/s41232-022-00236-4 (PMC9749184; doi:10.1186/s41232-022-00236-4)

**a**

Iba1/Hoechst

Control

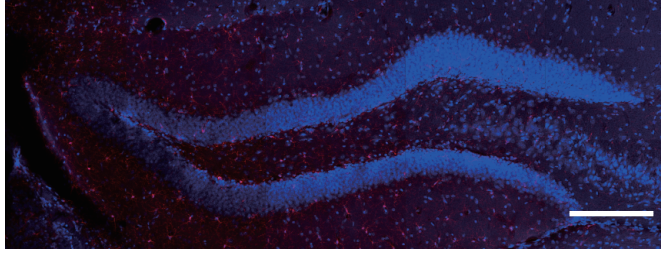

Sham

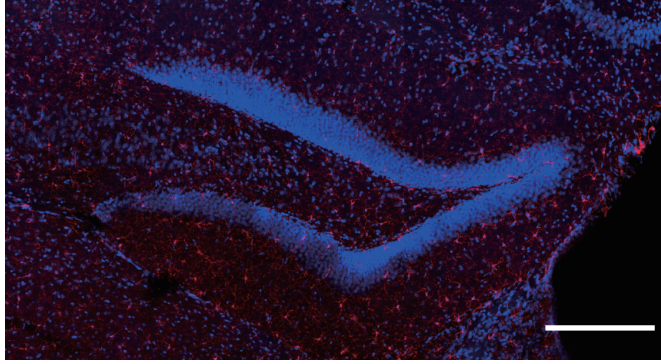

Mild SAH

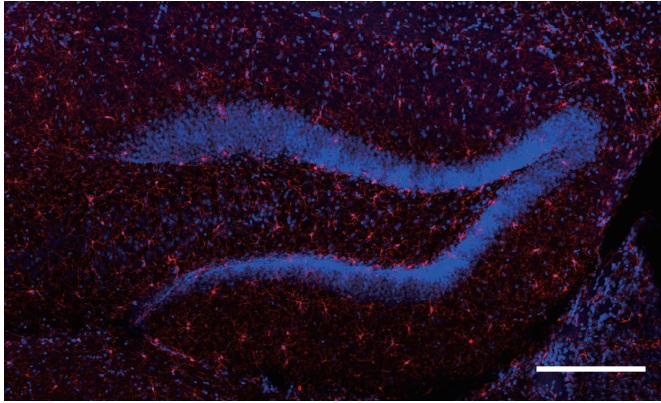

Severe SAH

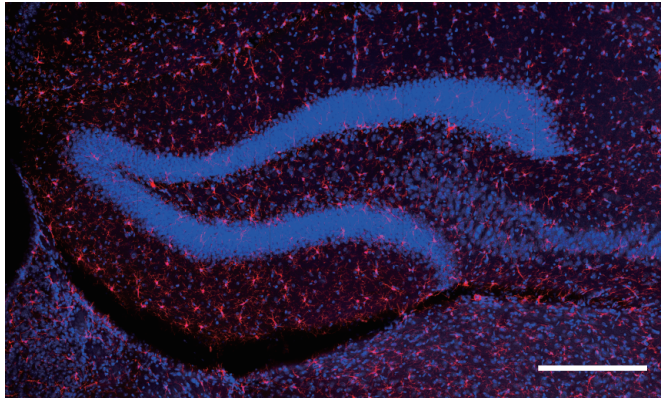

Figure S1

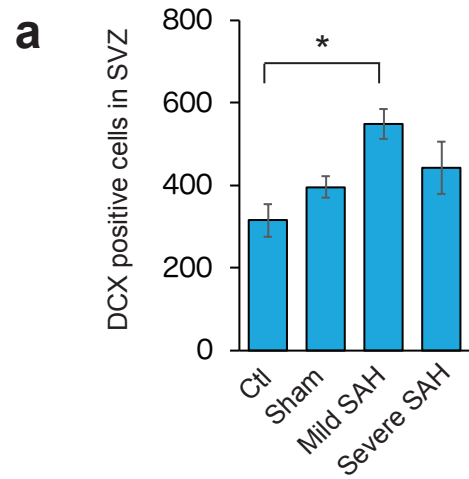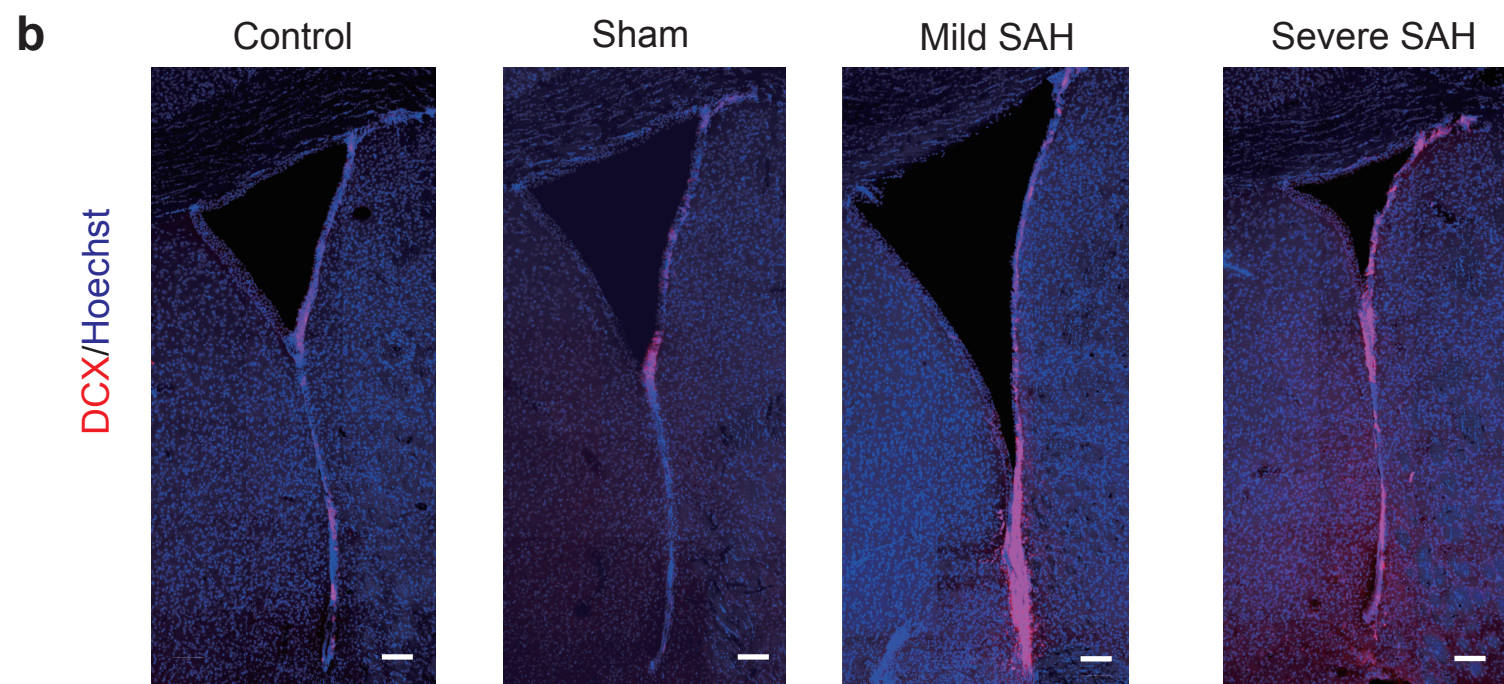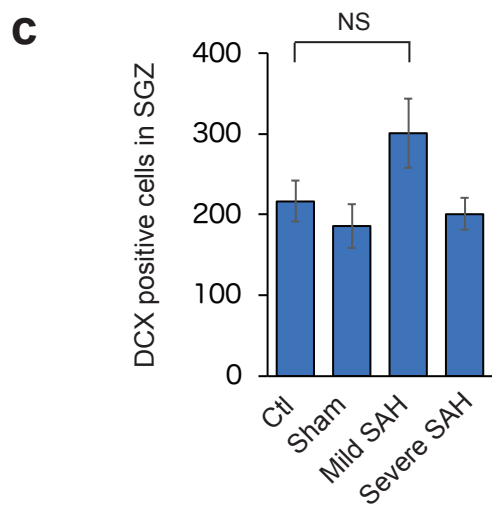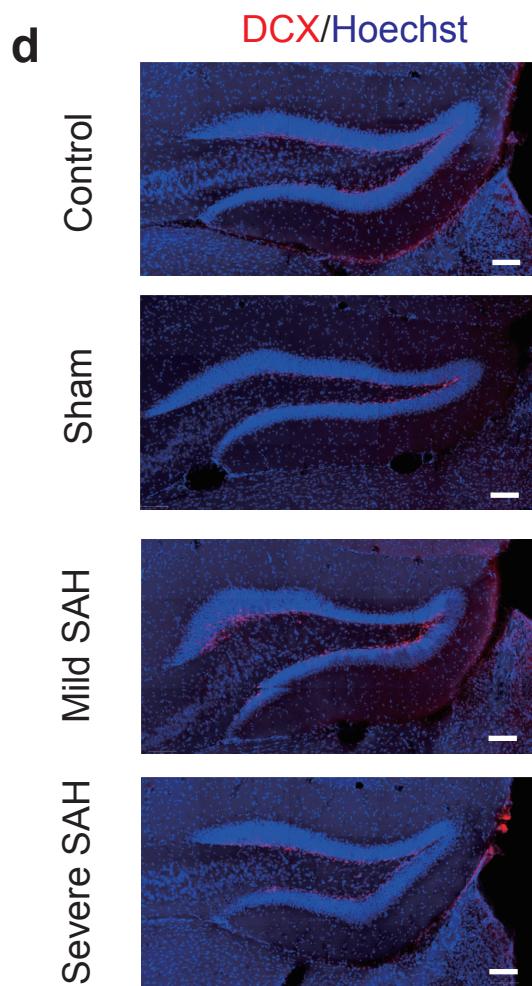

Figure S2

Supplement: Supplementary file 1 — Additional file 1. Supplementary Figure S1, Neuroinflammation in the hippocampus. (a) Confocal images of merged Iba 1 (red) and Hoechst (blue) in the overall view of the hippocampus in control, sham, mild SAH, and severe SAH mice. Scale bar, 100 μm. Supplementary Figure S2, Adult neurogenesis in the V-SVZ and SGZ. (a) Quantification of the number of DCX-positive cells in the V-SVZ (n=4, each group, control vs. mild SAH; p=0.022). (b) Representative confocal images of DCX (red) in the V-SVZ. Scale bar, 100 μm. (c) Quantification of the number of DCX-positive cells in the SGZ (n=4, each group, control vs. mild SAH; p=0.268). (d) Representative confocal images of DCX (red) in the SGZ. Scale bar, 100 μm. Statistical analyses were performed with one-way ANOVA and Tukey–Kramer post hoc tests. The values in the bar graphs represent the mean±SE. *p<0.05, NS; not significant. [file 41232_2022_236_MOESM1_ESM.pdf]
